# Prognostic significance of muc4 expression in gallbladder carcinoma

**DOI:** 10.1186/1477-7819-10-224

**Published:** 2012-10-27

**Authors:** Hyeon Kook Lee, Min-Sun Cho, Tae Hun Kim

**Affiliations:** 1 Department of Surgery, School of Medicine Ewha Womans University, 911-1 Mok-dong, Yangcheon-gu, Seoul, 158-710, South Korea; 2 Department of Pathology, School of Medicine Ewha Womans University, 911-1 Mok-dong, Yangcheon-gu, Seoul, 158-710, South Korea; 3 Department of Internal Medicine, School of Medicine Ewha Womans University, 911-1 Mok-dong, Yangcheon-gu, Seoul, 158-710, South Korea

**Keywords:** MUC1, MUC2, MUC4, Gallbladder carcinoma, Prognosis

## Abstract

**Background:**

Mucins are high molecular glycoproteins and play protective and lubricating roles in various epithelial tissues. Deregulated expression of mucins is involved in carcinogenesis and tumor invasion. MUC4 expression has been identified as a poor prognostic factor in pancreatobiliary carcinomas. To date, the relation between MUC4 expression and prognosis in gallbladder carcinoma remains to be determined. Authors examined MUC4 expression in gallbladder carcinoma and investigated its impact on prognosis.

**Methods:**

The expression profiles of MUC4, MUC1, MUC2 mucins in gallbladder carcinoma tissues from 63 patients were investigated using immunohistochemical staining.

**Results:**

For gallbladder carcinoma, positive staining of MUC4, MUC1, and MUC2 was 55.6%, 81.0%, 28.6%, respectively. There was a significant correlation between the expression of MUC4 and the expression of MUC1 or MUC2 (p = 0.004, p = 0.009, respectively). Univariate analysis showed that MUC4 expression (p = 0.047), differentiation (p < 0.05), T-stage (p < 0.05) and lymph node metastasis (p < 0.001) were significantly associated with poor survival. Expression of MUC1 and MUC2 was not correlated to survival. The backward stepwise multivariate analysis showed that MUC4 expression (p = 0.039) and lymph node metastasis (p = 0.001) were significant independent risk factors. In combined assessment of MUC4 and MUC2 expression, MUC4 positive and MUC2 negative group showed a significantly worse outcome than MUC4 negative groups(MUC4-/MUC2+ and MUC4-/MUC2-) and MUC4/MUC2 co-expression group(MUC4+/MUC2+) (p < 0.05).

**Conclusions:**

MUC4 expression in gallbladder carcinoma is an independent poor prognostic factor. Therefore, MUC4 expression may be a useful marker to predict the outcome of patients with surgically resected gallbladder carcinoma. MUC2 expression may have prognostic value when combined with MUC4 expression.

## Background

Gallbladder carcinoma still has a poor prognosis with 5-year overall survival rates at less than 15% [1,2]. Complete surgical resection for gallbladder carcinoma is the only potentially curative treatment option, but even in patients treated by curative resection, many of them showed poor outcome due to the high recurrence rate [3]. The commonly reported prognostic factors including pathologic stages are insufficient to predict either the clinical course or the biological behavior of gallbladder carcinoma. Therefore, investigation of new prognostic biomarkers is needed to predict the biological behavior of gallbladder carcinoma.

Mucins are high-molecular weight glycoproteins with oligosaccharides attached to serine or threonine residues of the mucin core protein backbone by O-glycosidic linkages. Mucins can be classified into two categories: transmembrane mucins (MUC1, MUC3, MUC4, MUC12, MUC13, MUC15, MUC16, MUC17, MUC20 and MUC21) and secreted mucins (MUC2, MUC5AC, MUC5B, MUC6, MUC7, and MUC19) [4-6]. The mucins are produced by various epithelial cells and serve protective and lubricating roles. However, in damaged epithelia or tumor cells, there is a loss of polarity that occurs in association with the activation of a proliferation and survival program [7]. Mucin gene expression is relatively organ-specific, and deregulated expression of one or more types of mucins occur with malignancy [8,9].

In pancreatobiliary neoplasms, MUC1 expression is related to invasive proliferation of tumors and/or a poor outcome for patients, whereas the expression of MUC2 is associated with noninvasive proliferation of tumors and a favorable prognosis [10-12].

MUC4 is a large transmembrane mucin first found in the tracheobronchial mucosa [13]. MUC4 is normally expressed in many epithelial tissues, including respiratory, colonic, and vaginal epithelia [14,15] and is overexpressed in various carcinomas such as breast, lung, ovarian, pancreatic [16-19]. Recently, MUC4 expression was revealed to be a significant prognostic factor for bile duct carcinoma and pancreatic ductal carcinoma [19-21]. However, little information is known about the prognostic role of MUC4 in gallbladder carcinoma, although gallbladder carcinoma is the most common malignancy in the biliary tract. Therefore, authors investigated MUC4 expression in gallbladder carcinoma by immunohistochemical staining to determine whether MUC4 expression could be a potential prognostic factor for gallbladder carcinoma.

## Methods

### Tissue sample

Surgically resected gallbladder carcinomas from 63 patients (17 male and 46 female patients) were studied retrospectively. The mean age of the patients was 66.9 years (range 36 to 88 years). The patients were diagnosed with gallbladder carcinoma and underwent operation between 1996 and 2006 in the Department of Surgery of Ewha Womans University Mokdong Hospital, South Korea. Resection with curative intent was performed in 53 patients (84%). Patients were selected for analysis based on the availability of paraffin-embedded tissue. Hematoxylin and eosin stained slides were reviewed for histological grade.

The pathological stage was classified according to the tumor node metastasis (TNM) classification of the American Joint Committee on Cancer Staging (AJCC) 6^th^ edition [22]. Clinicopathological data were reviewed and overall survival was analyzed. Follow-up was complete for 93% of enrolled patients. The follow-up period ranged from 1 to 122 months (mean, 36 months). This study was approved by the institutional review board of Ewha Womans University Mokdong Hospital.

### Immunohistochemistry

The primary antibodies used in this study were MUC1 (mouse monoclonal Ab NCL-MUC-1, Novocastra laboratories, Newcastle, UK), MUC2 (mouse monoclonal Ab NCL-MUC-2, Novocastra laboratories), MUC4 (mouse polyclonal antihuman MUC-4, Zymed, SF, CA, USA).

The resected tumors of patients who received surgery were fixed in 10% buffered formalin and embedded in paraffin wax. Sections of 4 μm thickness were mounted on gelatin dichromate-coated glass slides, deparaffinized and rehydrated through graded ethanol solutions to distilled water. Antigen retrieval was performed by heating slides in a microwave oven in 10 mM citrate buffer (pH 6.0). Immunohistochemical staining was then done using a semiautomated machine (Bond™ Automated Immunohistochemistry, Leica, Wetzlar, Germany) and the bond polymer detection system with counterstain (Leica). The process included endogenous peroxidase blocking by 0.3% hydrogen peroxide for 5 minutes, incubation with primary antibodies for MUC1, MUC2 and MUC4 at room temperature for 30 minutes, polymeric HRP-linker antibody conjugates as the secondary antibody, and expression using 3, 3^′^-diaminobenzidine. The expression of MUC1, MUC2 and MUC4 showed brown-colored cytoplasmic and membranous staining. The results of immnuohistochemical staining were considered to be positive if more than 5% of the neoplastic cells were stained. The percentages of positively stained neoplastic cells were graded as follows: +, > 5% but < 20% of neoplastic cells stained; ++, > 20% but < 50% of neoplastic cells stained; and +++, > 50% of neoplastic cells stained. When 5% or fewer neoplastic cells were stained, the results were considered to be negative.

### Statistical analysis

Statistical analysis was performed using the chi-square test, Fisher’s exact test, or the Spearman rank correlation test, where appropriate. Survival curves were plotted using the Kaplan-Meier method and differences between the survival curves were tested using the log-rank test. Multivariate survival analysis was performed using the Cox proportional hazards model. Backward stepwise multivariate analysis was also used to find independent prognostic factors. *P*-values less than 0.05 were considered statistically significant. All statistical analyses were conducted using SPSS 9.0 statistical software program (SPSS, Chicago, IL, USA).

## Results

### MUC1, MUC2, and MUC4 expression profile in gallbladder carcinoma

**Figure 1:**
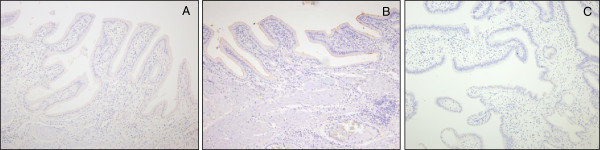
**Immunohistochemical staining of MUC4 (A), MUC1 (B), and MUC2(C) in normal gallbladder mucosa.** There was no expression of MUC4 and MUC2 in normal gallbladder epithelia, but weak expression of MUC1 along the apical membrane of the epithelium.

**Figure 2:**
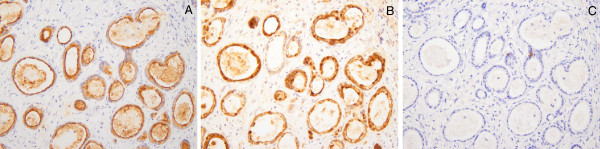
**Immunohistochemical staining for MUC4 (A), MUC1 (B), and MUC2 (C) in a patient with gallbladder carcinoma.** The expression of MUC4 and MUC1 was seen in the cytoplasm and/or membrane of the carcinoma cells, but expression of MUC2 was not seen.

Figure 1 shows the expression of the MUC4 (A), MUC1 (B), and MUC2 (C) in normal gallbladder tissue, while Figure 2 shows the expression of MUC4 (A), MUC1 (B), and MUC2 (C) in a patient with gallbladder carcinoma.

MUC 4 was expressed in 35 (55.6%) of the 63 gallbladder carcinomas. MUC1 was expressed in 51 (81.0%), and MUC2 was expressed in 18 (28.6%) carcinomas. There was significant correlation between the expression of MUC4 and the expression of MUC1 or MUC2 (*P* = 0.004, *P* = 0.009, respectively) (Table 1).

**Table 1:** **Correlation between MUC4**, **MUC2 and MUC1 expression in gallbladder carcinoma**

**MUC4 expression**					
	-	+	++	+++	*P*-value
MUC1 expression					
-	8	2	0	2	0.004
+	3	3	0	0	
++	5	8	0	1	
+++	12	3	8	14	
MUC2 expression					
-	24	7	5	9	0.009
+	4	2	2	5	
++	0	0	1	2	
+++	0	1	0	1	

Results are presented as the numbers of patients with carcinoma expressing MUC 4, and MUC1 or MUC2.

None of the expression of MUC1, MUC2, and MUC4 was significantly related to gender, age, differentiation, lymph node metastasis, or depth of invasion (Table 2).

**Table 2:** Relationships between mucin expression and clinicopathologic factors in patients with gallbladder carcinoma

	**MUC1**		**MUC2**		**MUC4**	
	**Number (%)**	***P*-value**	**Number (%)**	***P*-value**	**Number (%)**	***P*-value**
**Gender**						
Male (n = 17)	13 (77)	0.719^1^	2 (12)	0.116^1^	10 (59)	0.751^2^
Female (n = 46)	38 (83)		16 (35)		25 (54)	
**Age, years**						
< 65 (n = 26)	20 (77)	0.530^1^	7 (27)	0.808^2^	13 (50)	0.457^2^
≥ 65 (n = 26)	31 (84)		11 (30)		22 (60)	
**Differentiation**						
Well (n = 25)	19 (76)	0.517^1^	5 (20)	0.222^2^	11 (44)	0.134^2^
Moderate/poor (n = 38)	32 (84)		13 (34)		24 (63)	
**Lymph node metastasis**						
Negative (n = 28)	22 (79)	1.000^1^	11 (39)	0.128^2^	13 (46)	0.420^2^
Positive (n = 17)	14 (82)		3 (18)		10 (59)	
**Depth of invasion**						
T1/T2 (n = 34)	27 (79)	0.736^2^	10 (29)	0.873^2^	18 (53)	0.651^2^
T3/T4 (n = 29)	24 (83)		8 (28)		17 (59)	

^1^Assessed by Fisher’s exact test; ^2^assessed by the chi-square test.

### Prognostic significance of MUC4, MUC1, and MUC2 expression in gallbladder carcinoma

**Figure 3:**
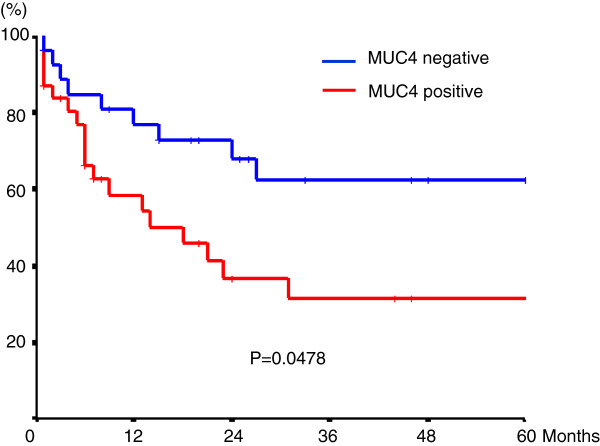
Survival curves according to the status of MUC4 expression in patients with gallbladder carcinoma.

Univariate analysis of factors related to overall survival in gallbladder carcinoma is summarized in Table 3. Patients with MUC4 expression had significantly worse survival than those without MUC4 expression (*P* = 0.048) (Figure 3). Other factors significantly correlated with survival were tumor differentiation, T stage, and lymph node metastasis. There was no significant difference in survival related to MUC1 expression. MUC2 expression was related to better survival but this was not statistically significant. The four factors (MUC4 expression, lymph node metastasis, T stage, and differentiation) selected from univariate analysis, based on a *P*-value < 0.05, were entered into the Cox proportional hazards model. This multivariate analysis showed that none of MUC4 expression (*P* = 0.079), lymph node metastasis (*P* = 0.080), T stage (*P* = 0.511), and differentiation (*P* = 0.291) were significant risk factors affecting the outcome (Table 4). In addition, backward stepwise multivariate analysis was used to find independent prognostic factors among these four factors listed in Table 4. Backward stepwise multivariate analysis showed that MUC4 expression (*P* = 0.039) and lymph node metastasis (*P* = 0.001) were statistically significant independent prognostic factors (Table 5).

**Table 3:** Univariate analysis of factors related to survival in patients with gallbladder carcinoma

**Factors**	**Patients alive at 5 years, %**	***P*-value**
**Age, years**		0.0986
< 5	60.8	
≥ 65	46.2	
**Gender**		0.9016
Male	42.9	
Female	47.9	
**Differentiation**		0.0013
Good	81.9	
Moderate/poor	25.2	
**MUC1 expression**		0.8882
Negative	37.3	
Positive	56.3	
**MUC2 expression**		0.1288
Negative	43.7	
Positive	56.3	
**MUC4 expression**		0.0478
Negative	62.3	
Positive	31.5	
**T stage**		0.0008
T1/T2	69.3	
T3/T4	19.9	
**LN metastasis**		0.0001
Negative	78.5	
Positive	23.3	

**Table 4:** Multivariate analysis of prognostic factors in patients with gallbladder carcinoma

**Factors**	**Risk ratio**	**95% CI**	***P*-value**
**MUC4**			
Negative	1		
Positive	2.890	0.884- 9.451	0.079
**Lymph node metastasis**			
Negative	1		
Positive	3.508	0.860- 14.308	0.080
**T stage**			
T1/T2	1		
T3/T4	1.626	0.381- 6.940	0.511
**Differentiation**			
Well	1		
Moderate/poor	2.589	0.443- 15.135	0.291

**Table 5:** Backward stepwise multivariate analysis of prognostic factors in patients with gallbladder carcinoma

**Factors**	**Risk ratio**	**95% CI**	***P*-value**
**MUC4 expression**			
Negative	1		
Positive	3.268	1.063 10.047	0.039
**Lymph node metastasis**			
Negative	1		
Positive	6.937	2.249 21.393	0.001

**Figure 4:**
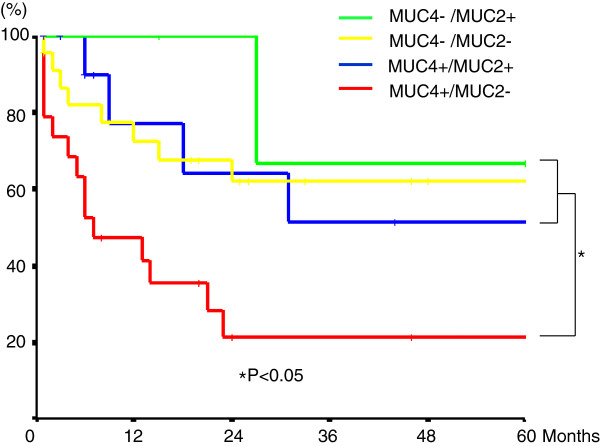
Survival curves according to the combined MUC4 and MUC2 expression in patients with gallbladder carcinoma.

In the combined assessment of MUC4 and MUC2 expression, the group with MUC4-positive and MUC2-negative pattern showed a worse outcome than the MUC4-negative groups (MUC4-/MUC2+ and MUC4-/MUC2-) and the MUC4/MUC2 co-expression group (MUC4+/MUC2+) (*P* < 0.05) (Figure 4).

## Discussion

Aberrant MUC4 expression has been observed in a variety of types of carcinoma. As to the biliary epithelial cells, MUC4 is not expressed in normal bile duct and gallbladder cells, but is expressed in cholangiocarcinoma cells [23,24]. MUC 4 expression has been reported to be a significant independent factor for poor prognosis in several cancers such as bile duct carcinoma, invasive ductal carcinoma of the pancreas, and small sized lung adenocarcinoma [19-21,25]. However, there has been no report on the prognostic role of MUC4 expression in gallbladder carcinoma. This study revealed for the first time that MUC4 expression in gallbladder carcinoma is an independent risk factor for poor outcome. This result suggests that MUC4 expression has an important role in predicting prognosis for patients with gallbladder carcinoma and has potential as a new molecular marker for clinical management of high risk patients. Given that this study shows the prognostic significance of MUC4 expression in gallbladder caricnoma, MUC4 expression should be considered a significant predictor for pancreato-biliary carcinomas including those of the gallbladder.

### MUC4 seems to use multiple mechanisms to engage pathways leading to tumor cell progression

MUC4 has been shown to function in cell growth signaling through interaction with the ErbB2 family of growth factor receptors and activation of ErbB2 downstream signaling pathways, resulting in cell proliferation and survival [14,26,27]. A recent study by Miyahara et al. showed that MUC4 is upregulated and interacts with ErbB2 in gallbladder carcinoma [28]. MUC4 can also promote tumor progression by repressing apoptosis by means of multiple mechanisms. The effects of MUC4 on conferring resistance to apoptosis have been found to be mediated by both ErbB2-dependent and ErbB2-independent mechanisms, indicating that tumor cells could exploit the versatile anti-apoptotic activities of MUC4 to acquire resistance to therapeutic agents, and augment cell survival [29]. Repression of MUC4 expression might be a therapeutic target for eliminating some of those resistance mechanisms. However, the molecular mechanisms underlying MUC4 anti-apoptotic activity have not been well characterized and need to be investigated.

There have been small numbers of studies addressing the profile of MUC expression in gallbladder carcinoma. However, they have focused on MUC1 and MUC2 expression and their association with clinicopathological factors. MUC1 is a transmembrane glycoprotein, and is the most intensively studied member of the mucin family, making MUC1 overexpression one of the more common alterations in human cancers [30]. Various studies showed that MUC1 expressed in tumors may function as an anti-adhesion molecule that inhibits cell-to-cell adhesion, permitting invasion into surrounding tissues [5,30,31]. MUC1 expression is significantly higher in gallbladder carcinoma than in normal and inflammatory gallbladder epithelium. Ghosh et al. and Yamato et al. reported MUC1 expression in 80% and 78.4% of gallbladder carcinoma, respectively, yet only traces in normal and inflammatory tissues [32,33]. This study also observed high expression of MUC1 (81%) in gallbladder carcinoma. These observations support the theory that MUC1 expression could be particularly prevalent in gallbladder carcinoma, and could contribute to the progression of gallbladder carcinomas. Regarding the prognostic value of MUC1 expression in gallbladder carcinoma, several studies described an association with a poor outcome in univariate analysis [34,35], but this study showed no relation between MUC1 expression and the outcome of patients with gallbladder carcinoma. This difference might be explained by various glycoforms of the MUC1 antigen, including underglycosylated, sialylated, and fully glycosylated forms [6], and these discrepancies in expression rates.

MUC2 is a gel-foaming secreted mucin that covers the intestinal mucosa, protecting the mucosal surface, and plays a role as lubricant. MUC2 has been found to be expressed mainly in the noninvasive tumors such as intraductal papillary mucinous neoplasm of the pancreas (IPMN) and mucin-producing bile duct tumor (MPBT) [10,36], whereas MUC2 does not appear to be expressed in pancreatic ductal adenocarcinoma [37]. High levels of MUC2 expression in indolent human pancreatobiliary neoplasms with a favorable prognosis may be related to the tumor suppressor activity of MUC2 [6].

The studies on MUC2 expression by gallbladder carcinoma showed that MUC2 expression is related to lower proliferative activity, contrary to MUC1 expression [33]. These results suggest that MUC2 expression is related to noninvasive proliferation of the tumor, resulting in a favorable outcome. But MUC2 expression has not been related to clinical outcome in gallbladder carcinoma [34,35]. This study also found that although MUC2 expression appeared linked to better survival, this was not statistically significant. These findings may be due to relatively low MUC2 expression in gallbladder carcinoma but further investigations are needed to clarify the mechanism of MUC2 expression.

This study found that MUC2 expression was correlated with MUC4 expression. When the combined status of MUC 4 and MUC2 expression was analyzed, MUC4-positive and MUC2-negative group was linked to a significantly worse outcome than in the MUC4-negative groups (MUC4-/MUC2+ and MUC4-/MUC2-) and the MUC4/MUC2 co-expression group (MUC4+/MUC2+). The point is that in MUC4-positive patients MUC2 expression status influences prognosis. These findings suggest that MUC2 expression may have prognostic value when combined with MUC4 expression.

## Conclusions

This study shows that MUC4 expression is an independent poor prognostic factor in gallbladder carcinoma. Therefore, MUC4 expression may be a useful marker to predict the outcome of patients with surgically resected gallbladder carcinoma. MUC2 expression may have prognostic value when combined with MUC4 expression.

## Competing interests

All authors declare no competing interests.

## Authors' contributions

HKL conceived and designed the study; HKL, MSC and THK acquired the data; HKL and MSC analyzed and interpreted the data; HKL prepared for the manuscript. HKL and THK edited the manuscript. All authors read and approved the final manuscript.
